# PREBIOTIC: a study protocol of a randomised controlled trial to assess prebiotic supplementation in kidney transplant recipients for preventing infections and gastrointestinal upset — a feasibility study

**DOI:** 10.1186/s40814-023-01236-y

**Published:** 2023-01-16

**Authors:** Samuel Chan, Carmel M. Hawley, Elaine M. Pascoe, Christopher Cao, Katrina L. Campbell, Scott B. Campbell, Ross S. Francis, Rachael Hale, Nicole M. Isbel, Mark Morrison, David W. Johnson

**Affiliations:** 1 Department of Nephrology, Princess Alexandra Hospital, Woolloongabba, Queensland Australia; 2 Australasian Kidney Trials Network, The University of Queensland, Brisbane, Queensland Australia; 3 Translational Research Institute, Brisbane, Queensland Australia; 4 The University of Queensland Diamantina Institute, Faculty of Medicine, University of Queensland, Woolloongabba, Queensland Australia

**Keywords:** Adherence, Clinical trial, Feasibility, Gut microbiota, Kidney failure, Kidney transplantation, Prebiotics, Recruitment, Tolerance

## Abstract

**Background:**

Modulating the microbiota in the large intestine of kidney transplant recipients through prebiotic supplementation may prevent infectious complications from occurring. To date, there have been no interventional trials which have investigated this novel treatment in kidney transplantation. The aim of PREBIOTIC is to assess the feasibility of performing a randomised controlled trial of prebiotics in reducing infections and gastrointestinal symptoms in kidney transplant recipients.

**Methods:**

Sixty kidney transplant patients will be recruited to a double-blind, placebo-controlled, randomised feasibility trial. Patients will be provided with prebiotic therapy or placebo for 4 to 6 weeks. Outcomes will include recruitment, adherence, tolerance, retention, laboratory parameters (including serum indoxyl sulphate, ρ-cresyl sulphate and stool collection), patients’ self-assessed quality of life, gastrointestinal symptoms and clinical outcomes.

**Discussion:**

This trial will assess the feasibility of prebiotic supplementation in kidney transplant recipients. Prebiotics not only may alter the gut microbiota and their inherent metabolism and production of uraemic toxins but also may prevent infections from occurring in kidney transplant recipients.

**Trial registration:**

Australian New Zealand Clinical Trials Registry number ACTRN12618001057279p. The date of registration was 25th June 2018, https://www.anzctr.org.au/Trial/Registration/TrialReview.aspx?id=375370&isReview=true.

## Introduction

### Background

Infections are a common complication following kidney transplantation, occurring in 40–65% of kidney transplant recipients in Australia and worldwide [1–3]. It is expected that the burden of infectious complications will continue to rise based on the growing prevalence of diabetes mellitus and the increasing potency of immunosuppressive regimens [4, 5]. It is therefore unsurprising that the Standardised Outcomes of Nephrology-Transplantation (SONG-Tx) initiative has identified infection as a core outcome that should be reported in all kidney transplant trials [6].

Over the last 5 years, emerging evidence has indicated that the gastrointestinal microbiota may play a role in the pathogenesis of infections in kidney transplant recipients [7–9]. The human microbiota are a collective of specialised communities of commensal, symbiotic and pathogenic micro-organisms (bacteria, archaea, fungi, protozoa and viruses) that colonise different sites and surfaces of the human body, of which the gastrointestinal tract has received the most intense investigation [10–12]. Various factors may influence the composition and diversity of the gastrointestinal microbiota including age, sex, medications, medical conditions and diet [13, 14]. Studies have indicated that gut dysbiosis in patients with chronic kidney disease may be associated with increased production of uraemic toxins, such as indoxyl sulphate (IS) and ρ-cresyl sulphate (pCS), which have been associated with intestinal inflammation, renal tubulointerstitial fibrosis and the progression of kidney disease [15, 16]. Gastrointestinal symptoms have also been shown to negatively impact quality of life in kidney transplant recipients [17].

Since the composition and diversity of the gastrointestinal microbiota may be modified by diet, it has been hypothesised that nutritional interventions, such as prebiotics (food sources that promote the growth of beneficial intestinal micro-organisms), probiotics (live micro-organisms that confer health benefits when ingested) and synbiotics (combined prebiotics and probiotics), may be a therapeutic opportunity to mitigate infectious complications in transplant recipients [13, 14, 18]. To the best of our knowledge, there have been no studies that have examined nutritional therapies in kidney transplant recipients. A meta-analysis of four studies (3 randomised controlled trials and 1 historically controlled trial) involving 246 liver transplant recipients has shown that administration of synbiotics resulted in appreciably lower rates of overall infection (relative risk [RR] 0.21, 95% *CI* 0.11–0.41, *I*^2^ = 1%), urinary tract infection (*RR* 0.14, 95% *CI* 0.04–0.47, *I*^2^ = 0%) and intra-abdominal infection (*RR* 0.27, 95% *CI* 0.09–0.78, *I*^2^ = 0%) [18]. The limitations of this review included moderate heterogeneity of the prebiotic and probiotic interventions, small sample sizes, short follow-up durations, restriction to only liver transplant recipients, inclusion of a non-randomised controlled trial and low certainty of evidence. Thus, there is an unmet need to assess the feasibility of establishing a randomised controlled trial in examining the effectiveness of prebiotic supplementation in preventing infections and gastrointestinal symptoms in kidney transplant recipients.

## Methods

### Aims and objectives

The aim of this study is to assess the feasibility of performing a randomised controlled trial of prebiotics in reducing infections and disruptive gastrointestinal symptoms in kidney transplant recipients. The primary objectives are to determine the following:

- This Prospective Randomised Evaluation of preBiotic In solid Organ Transplant recipients to prevent Infectious Complications (PREBIOTIC) study will be able to successfully recruit 60 patients within 6 months.
- Kidney transplant recipients will be adherent to prebiotic supplementation during the first 4 to 6 weeks following kidney transplantation.
- Prebiotic supplementation will be well tolerated by kidney transplant recipients during the first 4 to 6 weeks following kidney transplantation.
- Withdrawal of kidney transplant recipients will be minimal from a randomised controlled trial examining the safety and efficacy of prebiotic supplementation as an infection prevention strategy during the first 4 to 6 weeks following kidney transplantation.
- Kidney transplant recipients will be able to provide (in at least 80% of patients) two stool samples and two blood samples during the first 4 to 6 weeks following kidney transplantation to ascertain whether prebiotic supplementation improves dysbiosis.
- Prebiotic supplementation in kidney transplant recipients will result in improved quality of life during the first 4 to 6 weeks following kidney transplantation.
- Prebiotic supplementation in kidney transplant recipients will reduce the number of infections and infection-related hospitalisations during the first 4 to 6 weeks following kidney transplantation.

### Trial design

**Fig. 1:**
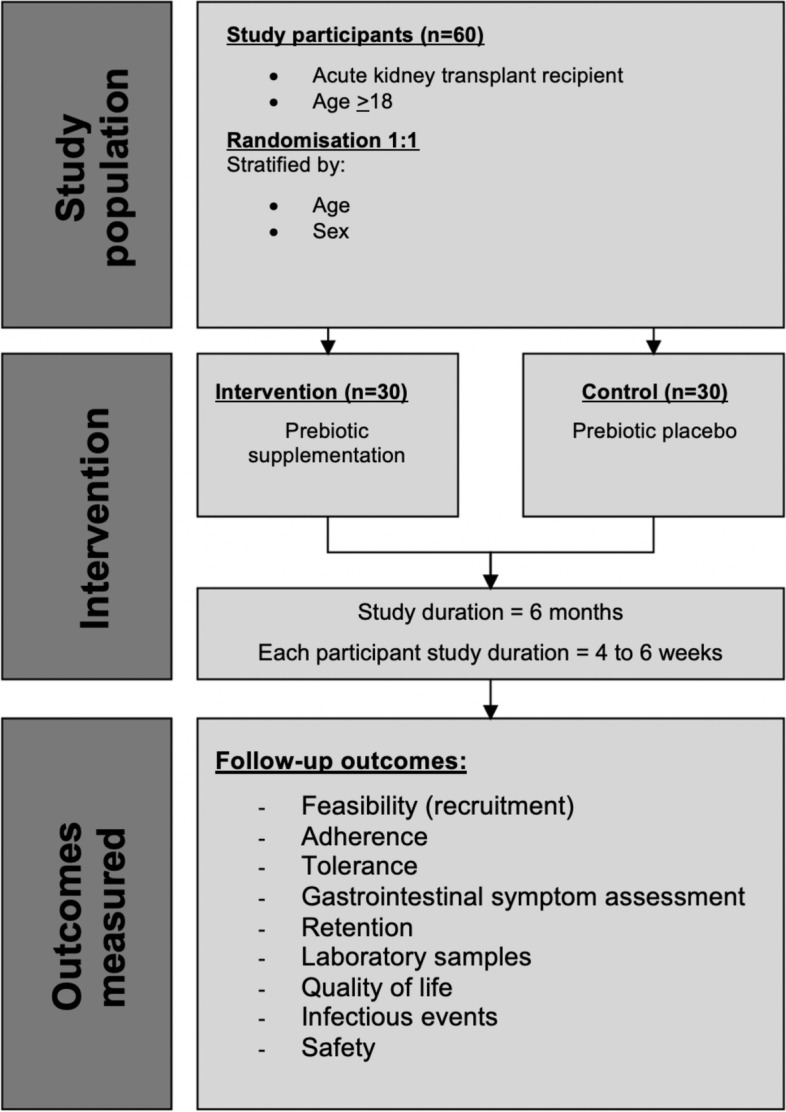
PREBIOTIC study schema

This study is a single-centre, parallel group, double-blind, randomised placebo-controlled feasibility trial (Fig. 1). Informed consent will be obtained from all participants. The study will be conducted according to the International Committee of Harmonisation (ICH) and good clinical practice (GCP) guidelines and reported according to CONSORT guidelines.

### Setting and participants

Sixty acute kidney transplant recipient patients will be recruited from the Princess Alexandra Hospital kidney transplant ward (Brisbane, Queensland, Australia) over a 6-month period. The Princess Alexandra Hospital is the only kidney transplant unit in Queensland and is the largest kidney transplant unit in the Oceania region. To be eligible to participate in this trial, participants must satisfy all of the following criteria: receive a kidney transplant at the Queensland Renal Transplant Service, be aged ≥ 18 years and be able to provide informed consent. Patients will be excluded from participation if they meet any of the following criteria: have received radiation to the bowel and/or large bowel resection, medically diagnosed and active inflammatory bowel disease, unwilling or unable to meet the requirements of the protocol and other medical, social, cultural and/or linguistic reasons negatively affecting their adherence to the protocol, at the discretion of the investigators.

### Participant identification and recruitment process

Patients will be provided with written and verbal information about the study while an inpatient in the transplant ward. They will be given the opportunity to ask questions, to take the information home with them when they leave hospital and to discuss it with friends, family or others. They will be able to provide consent at one of their routine clinic visits in the early post-discharge period. This will ensure that the patients are physically and mentally well and stable and thereby able to fully consider their participation in the study. Once they have consented to participate in the study (usually between day 5 to day 12 following acute kidney transplant), participants will commence the prebiotic powder or placebo suspended in water daily for 4 to 6 weeks. The initial dosage will be 7.5 g daily for the first 2 weeks, thereafter increasing to 15 g daily for the final 2 to 4 weeks of the study.

### Treatment

This trial will consist of two arms: (1) green banana-resistant starch (prebiotic supplement) and (2) waxy maize (matched, identical placebo).

#### Intervention — green banana-resistant starch

The active intervention in this study will be green banana-resistant starch multi-fibre. This is a functional food product structurally resistant to digestion in the small intestine [19]. When resistant starches reach the large intestine, the polymers can be deconstructed and fermented by the resident microbiota. The beneficial effects from these products include a laxative effect promoting regularity of bowel motions and the capacity to promote the growth of select commensal microbes, in addition to beneficial effects reported on lipid-based biomarkers in subjects with type 2 diabetes mellitus.

#### Comparator — waxy maize

The comparator (control) in this study will be waxy maize, which is primarily comprised of branched starch polymers (amylopectin) that are more readily digestible in the small intestine. As such, much of the placebo should be digested proximal to the large intestine and will have limited impacts on the large intestinal microbiota.

### Concomitant treatment

All other treatment, including medications, will be as per standard care for the patient.

### Randomisation

Participants will be randomly assigned in a 1:1 ratio to receive either prebiotic or placebo. The randomisation schedule will be prepared by a researcher not involved with treatment allocation. Standard blocking and stratification will be used to ensure between-group balance of size and patient characteristics, including age (< 65 years, ≥ 65 years) and sex. A blinded allocation list will be maintained in an Excel spreadsheet on a secure server not accessible to staff involved in study recruitment and data collection.

### Blinding

Participants, caregivers, treating physicians and surgeons, laboratory staff and members of the study team will be blinded to the treatment. Only the statistician not involved with recruiting patients in this study will be aware of the product allocation sequence.

### Primary and secondary outcomes

The primary outcome of this study is feasibility of recruitment which will be defined as at least 80% of eligible subjects recruited to the study.

The secondary outcomes will include the following:

- Timeliness of recruitment

This will be assessed as the ability to successfully recruit 60 patients within 6 months.

b) Adherence

This will be assessed as the proportion of participants adherent to prescribed study therapy (intervention or placebo) over the period of the study. Adherence will be defined as having used 80% of more of the prescribed study therapy, calculated by the % of the expected weight of the product returned divided by the initial weight of study therapy.

iii) Tolerance

This will be assessed as the proportion of patients who continue the prebiotic supplementation. Tolerance will be defined as 80% of the recruited patients taking the prescribed study therapy during the study period.

iv) Gastrointestinal symptom assessment

The gastrointestinal symptom assessment will be assessed as the changes in the Gastrointestinal Symptom Rating Scale score from baseline compared with the score at the time of the completion of the PREBIOTIC trial.

e) Retention

This will be assessed as the proportion of patients who remain in the PREBIOTIC study for the entire study period.

f) Laboratory samples

There will be two aspects to this outcome. The first part will examine the proportion of participants providing two stool samples at designated times (during the first week and between 4 and 6 weeks post-kidney transplant) with sufficient material to assess gut microbiota (stool sample analysis via shotgun metagenomic sequencing to a target depth of 3 Gbp using NovaSeq 6000, 2 × 150 base pair, paired-end chemistry). The second part of this outcome will assess the proportion of participants providing two blood samples for serum indoxyl sulphate and p-cresyl sulphate measurement at designated times (during the first week and between week 4 to week 6 post-kidney transplant).

g) Quality of life

This will be assessed as the changes in the overall quality of life score (measured by EQ-5D survey) from baseline compared with the score measured at the completion of the PREBIOTIC trial.

h) Infectious events

This will be assessed as the proportion of patients who develop at least one infectious event requiring hospital admission or antimicrobial therapy. Infectious adverse events of special interest would include the following:

- Urinary tract infections
- Gastrointestinal infections
- Respiratory infections (e.g. community or hospital acquired)
- Opportunistic infections (e.g. cytomegalovirus, BK virus)
- Skin or soft tissue infections
- Central nervous system infections

i) Safety

A serious adverse event (SAE) will be defined as any event/reaction that results in death, is life-threatening, requiring hospitalisation or prolongation of exisiting hospitalisation and resulting in persistent or significant disability or incapacity. All SAEs will be documented and reported to the ethics committee for review.

Prebiotic supplementation may also increase serum potassium particularly during the early days following kidney transplantation. The hyperkalaemia may also be related to constipation from prebiotic supplementation. Nevertheless, the participant’s serum potassium will be checked as per usual clinical routine (daily for the first 3 weeks then thrice weekly thereafter). Patients may be advised to stop the prebiotic temporarily if their serum potassium levels increase above 6.0 mmol/L.

### Data collection

Data will be entered into Microsoft Excel during the course of the feasibility study, and data will be recorded only by investigators of the study. This system will help ensure compliance with medical data privacy, security and good clinical practice regulations. Data will be stored in password-protected files for 15 years and then destroyed. Physical copies of data will be kept in a locked filing cabinet. The data to be collected in this study are depicted in Table 1.

**Table 1:** Data collection schedule and parameters to be collected

		Wk0	Wk2	Wk4	Wk6
**Patient information**	Consent and patient demographics	X			
	Medical history	X			
	Medications	X	X	X	X
	Infection risk	X			
	Immunological risk	X			
	Transplant history	X			
	Transplant course specifically documenting infection events		X	X	X
**Standard care biochemistry**	Full blood count Urea and electrolytes Fasting blood glucose HbA1c	X	X	X	X
**Uremic toxins**	Total serum IS	X			X
	Free serum IS	X			X
	Total serum pCS	X			X
	Free serum pCS	X			X
**Gut microbiota**	Bristol stool evaluation and stool sample collection	X	X		X
**Inflammatory markers**	C-reactive protein	X			X
**Quality of life (EQ-5D)**		X			X
**Prebiotic dose commencement**		X	X		
**Prebiotic dose escalation**			X		
**Gastrointestinal symptoms (GSRS)**		X	X		X
**Compliance**	Weight of powder				X
**Serious adverse events**			X	X	X
**Adverse events**			X	X	X

### Sample size

A sample size of 60 subjects will be required for an estimate of 95% confidence interval to within plus or minus 11%, assuming a recruitment rate of 80%.

### Planned analysis

As this is a feasibility study, data will be analysed via descriptive statistics, expressing frequencies (percentages) for categorical data, mean ± standard deviation for continuous normally distributed data or median (interquartile range) for continuous non-normally distributed data. In addition, confidence intervals will be presented for all descriptive statistics. Outcome measures and the corresponding statistical measures are shown in Table 2. Analysis will be performed on an intention-to-treat basis. Patients discontinuing the study drug for whatever reason will be encouraged to continue follow-up in the trial. The null hypothesis will be rejected at the 0.05 level. The statistical analyses will be performed using Stata (version 14, 2016, Statacorp, College Station, TX, USA).

**Table 2:** Outcomes and corresponding measures

Outcome	Measure
Proportion of eligible patients who agree to take part in the study	% (95% CI)
Ability to successfully recruit 60 patients within 6 months	% (95% CI)
Proportion of participants adherent to prescribed study therapy (intervention or placebo) over the period of the study	% (95% CI)
Proportion of patients who continue to the prebiotic supplementation	% (95% CI)
Mean changes in the Gastrointestinal Symptom Rating Scale	Mean change (SD)
Proportion of patients who withdraw from the PREBIOTIC study	% (95% CI)
Proportion of participants providing two stool samples at designated times (the first week and between week 4 to week 6 post-kidney transplant) to assess gut microbiota changes	% (95% CI)
Proportion of participants providing two blood samples at designated times (the first week and week 6 post-kidney transplant)	% (95% CI)
Changes in the overall quality of life (measured by EQ-5D survey)	Mean change (SD)
Proportion of patients with at least one infectious event	% (95% CI)

### Ethical considerations

Ethical approval has been granted through the Metro South Human Research Ethics Committee (HREC/2020/QMS/51887) and The University of Queensland Human Research Ethics Committee (51887).

### Trial governance

The Trial Management Group (TMG) comprising chief investigator and coinvestigators will provide overall management of the study including clinical set-up and training, centre set-up in preparation for recruitment, promotion of the study and interpretation of the results.

## Discussion

This double-blind placebo-controlled randomised study has been designed to assess the feasibility of a randomised controlled trial to examine whether prebiotic supplementation may prevent infections and gastrointestinal upset in kidney transplant recipients. A range of outcomes will be assessed including feasibility, adherence, tolerance, retention, laboratory testing, consumer-centred outcomes and clinical outcomes. It is imperative that a feasibility study is performed initially prior to embarking on a formal randomised controlled trial. First, a feasibility study is an important prerequisite for designing large-scale trials when critical factors important to the study design are unknown [20, 21]. Such unknown factors relevant to PREBIOTIC include the proportion of patients who meet eligibility criteria, have comorbid illnesses or choose to discontinue study medication. Second, a feasibility study should have clear aims [21], and for the PREBIOTIC feasibility study, these are to determine whether 60 patients can be recruited over a 6-month period as well as assess the proportion of eligible patients who agree to take part in the study. Third, it is important to note whether kidney transplant recipients will take green banana-resistant starch over a sustained period, because if adherence, tolerance and retention are poor, a formal randomised controlled trial would be critically compromised. Kidney transplant recipients already experience a considerable medication burden, and thus, assessment of adherence will help to better inform whether green banana-resistant starch is a suitable additional long-term supplement. Fourth, a feasibility study will allow investigators to establish a realistic timeline to ensure that the trial is completed in a timely manner [20, 21]. Validating a prospective timeline is an advantage of a feasibility study and may allow potential delays or breakdowns to be evaluated and clinical outcomes to be appropriately assessed.

Other outcomes which will be explored in this study include patient-centred factors such as quality of life and the patient’s symptoms with respect to their gastrointestinal health. A 6-month prospective, randomised, double-blind, placebo-controlled crossover trial of probiotic bacterial formulation involving 46 participants with stages 3 or 4 chronic kidney disease from four countries reported significant improvements in quality of life (86%, *p* < 0.05) [22]. However, there are no studies that have examined the impact of prebiotic supplementation on quality of life in patients with kidney disease. Furthermore, this feasibility study will include a gut microbiota analysis evaluating whether prebiotic strains alter the microbiota of the large bowel, as has been shown in infants [23] and influence serum concentrations of the putative uraemic toxins, indoxyl sulphate and serum *ρ*-cresyl sulphate [24].

The use of green banana-resistant starch in this feasibility study is a promising nutritional intervention since the functional properties may enhance short chain fatty acid production without triggering irritable bowel syndrome symptoms, which fermentable oligosaccharides, disaccharides, monosaccharides and polyols (FODMAPS) are known to do [25, 26]. Short-chain fatty acids, primarily acetate, propionate and butyrate are known to lower intestinal pH, which in turn can inhibit the growth of pathogenic bacteria [27, 28] and promote favourable metabolic effects leading to a reduction in the incidence of metabolic syndrome and improved insulin sensitivity [29, 30].

In summary, this PREBIOTIC study aims to provide proof-of-concept data to elucidate whether altering the gastrointestinal microbiota in the kidney transplant population is likely to be effective, tolerable and prevent infectious complications. If this feasibility study is shown to be successful, it will be used to inform the design and conduct of a large scale, multicentre randomised controlled trial.

## Data Availability

De-identified individual participant data that underlie the results reported in this publication can be requested by any qualified researchers. Medicare and all other administrative data will not be available. Methodologically sound proposals should be directed to aktn@uq.edu.au. The Australasian Kidney Trials Network Data Sharing Committee will assess proposals based on the following criteria: sound science, benefit-risk balancing and research team expertise. The data will be available in a digital repository supported by The University of Queensland but without investigator support other than deposited metadata. To gain access, data requestors will need to sign a data access agreement. Data will be available beginning 2 years after the publication of all pre-specified analyses.

## Abbreviations

PREBIOTIC: Prospective Randomised Evaluation of preBiotic supplementation In sOlid organ Transplant recipients to prevent Infectious Complications
RR: Relative risk
IS: Indoxyl sulphate
pCS: ρ-Cresyl sulphate

## References

[1] United States Renal Data System (2013). Annual data report: atlas of chronic kidney disease and end-stage renal disease in the United States.

[2] Methven S, Steenkamp R, Fraser S (2017). UK renal registry 19th annual report: chapter 5 survival and causes of death in UK adult patients on renal replacement therapy in 2015: national and Centre-specific analyses. Nephron..

[3] Australian Bureau of Statistics (2017). Causes of death, 2016- disease of the kidney, urinary system and genitals (N00-N99).

[4] Fishman JA (2017). Infection in organ transplantation. Am J Transplant.

[5] Wolfe RA, Roys EC, Merion RM. Trends in organ donation and transplantation in the United States, 1999–2008. Am J Transplant. 2010;10, 961(4p2):–72. 10.1111/j.1600-6143.2010.03021.x 20420646

[6] Fricke WF, Maddox C, Song Y, Bromberg JS (2014). Human microbiota characterization in the course of renal transplantation. Am J Transplant.

[7] Lee J, Magruder M, Zhang L, Westblade L, Satlin M, Robertson A (2019). Gut microbiota dysbiosis and diarrhea in kidney transplant recipients. Am J Transplant.

[8] Lee JR, Muthukumar T, Dadhania D, Toussaint NC, Ling L, Pamer E (2014). Gut microbial community structure and complications following kidney transplantation: a pilot study. Transplantation..

[9] Shreiner AB, Kao JY, Young VB (2015). The gut microbiome in health and in disease. Curr Opin Gastroenterol.

[10] Turnbaugh PJ, Ley RE, Hamady M, Fraser-Liggett CM, Knight R, Gordon JI (2007). The human microbiome project. Nature..

[11] Cho I, Blaser MJ (2012). The human microbiome: at the interface of health and disease. Nat Rev Genet.

[12] Ouwehand AC, Salminen S, Arvola T, Ruuska T, Isolauri E (2004). Microbiota composition of the intestinal mucosa: association with fecal microbiota?. Microbiol Immunol.

[13] Falony G, Joossens M, Vieira-Silva S, Wang J, Darzi Y, Faust K (2016). Population-level analysis of gut microbiome variation. Science..

[14] Zhernakova A, Kurilshikov A, Bonder MJ, Tigchelaar EF, Schirmer M, Vatanen T (2016). Population-based metagenomics analysis reveals markers for gut microbiome composition and diversity. Science..

[15] Nataatmadja M, Cho Y, Campbell K, Johnson DW. The roles of indoxyl sulphate and p-cresyl sulphate in patients with chronic kidney disease: a review of therapeutic options. Chronic Kidney Disease-from Pathophysiology to Clinical Improvements: IntechOpen; 2017.

[16] Rossi M, Johnson DW, Morrison M, Pascoe EM, Coombes JS, Forbes JM (2016). Synbiotics easing renal failure by improving gut microbiology (SYNERGY): a randomized trial. Clin J Am Soc Nephrol.

[17] Chan S, Cao C, Pascoe EM, Johnson DW, Shah A, Holtmann GA (2020). Patient-reported gastrointestinal symptoms and the association with quality of life following kidney transplantation.

[18] Sawas T, Al Halabi S, Hernaez R, Carey WD, Cho WK (2015). Patients receiving prebiotics and probiotics before liver transplantation develop fewer infections than controls: a systematic review and meta-analysis. Clin Gastroenterol Hepatol.

[19] Faisant N, Buléon A, Colonna P, Molis C, Lartigue S, Galmiche JP (1995). Digestion of raw banana starch in the small intestine of healthy humans: structural features of resistant starch. Br J Nutr.

[20] Lancaster GA, Thabane L (2019). Guidelines for reporting non-randomised pilot and feasibility studies.

[21] Thabane L, Hopewell S, Lancaster GA, Bond CM, Coleman CL, Campbell MJ (2016). Methods and processes for development of a CONSORT extension for reporting pilot randomized controlled trials. Pilot Feasibility Stud.

[22] Ranganathan N, Ranganathan P, Friedman EA, Joseph A, Delano B, Goldfarb DS (2010). Pilot study of probiotic dietary supplementation for promoting healthy kidney function in patients with chronic kidney disease. Adv Ther.

[23] Hasan N, Yang H (2019). Factors affecting the composition of the gut microbiota, and its modulation. PeerJ..

[24] Lin C-J, Wu V, Wu P-C, Wu C-J (2015). Meta-analysis of the associations of p-cresyl sulfate (PCS) and indoxyl sulfate (IS) with cardiovascular events and all-cause mortality in patients with chronic renal failure. PLoS One.

[25] Halmos EP, Christophersen CT, Bird AR, Shepherd SJ, Gibson PR, Muir JG (2015). Diets that differ in their FODMAP content alter the colonic luminal microenvironment. Gut..

[26] Halmos EP, Gibson PR (2019). Controversies and reality of the FODMAP diet for patients with irritable bowel syndrome. J Gastroenterol Hepatol.

[27] Esgalhado M, Kemp JA, Damasceno NRT, Fouque D, Mafra D (2017). Short-chain fatty acids: a link between prebiotics and microbiota in chronic kidney disease. Future Microbiol.

[28] McLoughlin RF, Berthon BS, Jensen ME, Baines KJ, Wood LG (2017). Short-chain fatty acids, prebiotics, synbiotics, and systemic inflammation: a systematic review and meta-analysis. Am J Clin Nutr.

[29] Kassaian N, Feizi A, Aminorroaya A, Jafari P, Ebrahimi M, Amini M (2018). The effects of probiotics and synbiotic supplementation on glucose and insulin metabolism in adults with prediabetes: a double-blind randomized clinical trial. Acta Diabetol.

[30] Maki KC, Pelkman CL, Finocchiaro ET, Kelley KM, Lawless AL, Schild AL (2012). Resistant starch from high-amylose maize increases insulin sensitivity in overweight and obese men. J Nutr.

